# Feasibility, diagnostic performance and clinical value of an abbreviated echocardiography protocol in an out-patient cardiovascular setting: a pilot study

**DOI:** 10.1186/s44156-022-00009-2

**Published:** 2022-09-15

**Authors:** Sher May Ng, Danial Naqvi, Jose Bingcang, Gemma Cruz, Richard Nose, Guy Lloyd, Marie Elsya Speechly-Dick, Sanjeev Bhattacharyya

**Affiliations:** 1 Department of Cardiology, University College London Hospital, London, UK; 2 Echocardiography Laboratory, University College London Hospital, 250 Euston Road, London, UK; 3 Echocardiography Laboratory, St Bartholomew’s Hospital, London, UK

**Keywords:** Abbreviated echocardiography, Chest pain clinic, Low risk outpatient

## Abstract

**Background:**

There has been a growing demand for echocardiography services over the last 5 years, with this supply–demand mismatch exacerbated by the COVID-19 pandemic. Prior studies have suggested a high proportion of normal findings among echocardiograms requested for patients without known cardiovascular disease, particularly in low-risk cohorts. This pilot study investigates the role of an abbreviated echocardiography protocol in improving access to echocardiography services in a low-risk outpatient setting within the rapid access chest pain (RACP) clinic.

**Method:**

A retrospective review of electronic medical records and transthoracic echocardiography (TTE) studies for 212 patients from RACP clinic in 2019 (cohort A), prior to the introduction of the abbreviated echocardiography protocol, and 175 patients seen in the RACP clinic in 2021 (cohort B) was performed. The outcomes measured include the echocardiography referral burden from RACP clinic, waiting time for a TTE and echocardiography findings.

**Results:**

33% and 45% of patients seen in the RACP clinic in 2019 and 2021, respectively, were referred for a TTE. The most common indications include chest pain (50%), dyspnoea (19%) and palpitations (11%). Abnormal findings were identified in 36% of TTEs performed in cohort A and 13% in cohort B. The median echocardiogram study time was significantly shorter in cohort B (7 min vs 13 min, p < 0.00001), with a lower number of images acquired (43 vs. 62, p < 0.00001). The median waiting time for an echocardiography in cohort B was significantly shorter (median: 14 days vs. 42 days in 2019, p < 0.00001). No major pathologies were missed on a retrospective review of these images.

**Conclusion:**

Our study demonstrates that an abbreviated echocardiography protocol has potential to improve access to echocardiography services through increasing scheduling capacity, without compromising diagnostic performance in a low-risk outpatient population.

**Supplementary Information:**

The online version contains supplementary material available at 10.1186/s44156-022-00009-2.

## Background

Chest pain accounts for between 1 and 3% of general practice (GP) consultations, with approximately 40% of patients referred to secondary care for further investigation [1]. Patients presenting with acute chest pain account for 2–6% of accident and emergency (A&E) attendances and up to 20% of hospital admissions UK-wide, with an estimated cost-burden to the National Health Service (NHS) of nearly £700 million (1.3% of total NHS expenditure) [2–4].

The introduction of rapid access chest pain (RACP) clinics in 2000 is seen as an effective and efficient alternative to outpatient general cardiology clinics for patients with new-onset chest pain, reducing the waiting time for patients with new-onset chest pain and potentially life-threatening coronary artery disease (CAD) [5]. A transthoracic echocardiography (TTE) may be required for evaluation of these patients where symptoms or physical findings suggest alternative diagnoses.

Echocardiography services across the UK have seen a steady increase in referrals in the 5 years prior to the COVID-19 pandemic. Approximately 1.6 million echocardiograms were performed across the UK between 2018 and 2019 with more than 4% of patients waiting for more than 6 weeks [6]. This supply–demand mismatch was made worse during the pandemic with cancellations of routine outpatient echocardiography lists. Despite efforts to improve the backlog of echocardiograms, latest published figures continue to show that the number of people in England waiting more than 6 weeks for an echocardiogram is ten times higher than before the pandemic [7].

An abbreviated echocardiography reduces the number of study images acquired by a level 2 or 3 qualified physiologist using a departmental, full functioning ultrasound machine. Whilst the initial protocol uses a limited number of images, the physiologist has the skills and knowledge to acquire additional images, views and quantification to answer specific clinical questions, or if certain abnormalities are identified [8]. This differentiates abbreviated echocardiography from focussed echocardiography or level 1 scans, which use fixed protocols with a defined scope of practice. The latter often do not include spectral Doppler and provide limited quantification of pathology [9]. The American Society of Echocardiography has published limited echocardiographic protocols for left ventricular function, pericardial effusion and pulmonary hypertension [10].

We hypothesised that a full protocol was not required in all patients requiring cardiovascular assessment particularly where the likelihood of the study being normal was high. Protocols limited to a single pathology e.g. left ventricular function would however, be inadequate. Therefore, we propose that access to echocardiography can be improved without missing major pathology through an abbreviated echocardiography protocol, where an experienced performing physiologist can proceed to investigate any abnormalities identified with further views or measurements as per a full study. In this study, we report our experience of an abbreviated echocardiography in an outpatient RACP clinic setting.

## Methods

A retrospective review of electronic medical records was performed for 212 patients seen in the RACP clinic between May and October 2019 (cohort A—full echocardiography studies) and 175 patients between May to October 2021 (Cohort B—abbreviated echocardiography studies). Patient demographics, cardiovascular risk factors, indications for echocardiography, time between referral to echocardiography and examination, as well as echocardiography findings were identified from the electronic patient record. Abbreviated studies were requested by the RACP clinic from May 2021 onwards if there was no known history or low likelihood of heart failure or valve disease. The study was approved by the NHS Trust as a clinical service evaluation.

All echocardiograms (full and abbreviated studies) were performed by level 2 British Society of Echocardiography (BSE)-accredited physiologists using commercially available ultrasound machines (General Electric Vivid E95). Full studies were performed according to the BSE minimum dataset [11]. Abbreviated studies were performed according to a defined minimum dataset specific to the abbreviated protocol (Additional file 1). Red flags (suspected valve stenosis, more than mild valve regurgitation, pericardial effusion, cardiac masses, aortic pathology, enlarged left atrium or left ventricular systolic impairment) were triggers for additional images and analysis. In these cases, the physiologists performed extra views and acquired additional images and quantification as per the comprehensive minimum BSE dataset. For example, if mitral regurgitation was detected, the degree of regurgitation was quantified using an integrated approach. All echocardiograms were reviewed and assessed by the department lead for adherence to the minimum dataset as well as whether any pathology was not identified, or inaccurately reported. This included the grade of left ventricular function, presence and grade of valvular disease, left atrial size and the presence of pericardial effusion.

For cohort A, a 45-min appointment was scheduled for the full echocardiography study. In a single half day session, 5 studies were booked. For cohort B, a 30-min appointment time was scheduled, with 8 studies booked in a half-day session.

The waiting time for a TTE was defined as that between the date of clinic appointment and the date the TTE was performed. The scan time was defined as the time between the acquisition of the first and last images.

Descriptive data are presented as number and percentage or median and inter-quartile range. Comparison of categorical and non-parametric numerical data were performed using the Chi-squared and Mann–Whitney U tests, respectively. All statistical analysis was performed using SPSS version 28. Statistical significance was defined as a p-value of < 0.05.

## Results

A total of 212 patients in cohort A and 175 in cohort B were identified. The baseline demographics of patients seen are summarised in Table 1. 47% and 51% of the total study cohort were treated for hypertension and hyperlipidaemia, respectively. Nearly a third of patients in cohort A were obese. There was low prevalence of prior coronary artery disease. The majority of referrals in both cohorts originated from primary care.

**Table 1:** Baseline demographics of cohort A and cohort B

		Cohort A (n = 212)	Cohort B (n = 175)	Significance (p-value)
Age (Median, years)		59	59	
Male		114 (54%)	92 (53%)	0.81
Ethnicity	Caucasian	130 (61%)	76 (43%)	0.0004
	Indian	36 (17%)	26 (15%)	0.57
	Chinese	5 (2%)	2 (1%)	0.37
	Black	18 (8%)	12 (7%)	0.55
	Mixed	3 (1%)	3 (2%)	0.81
	Other/Unknown	20 (9%)	56 (32%)	< 0.001
Cardiovascular Risk Factors	Current smoker	36 (17%)	30 (17%)	0.97
	Hypertension	94 (44%)	87 (50%)	0.29
	Hyperlipidaemia	97 (46%)	101 (58%)	0.02
	Diabetes Mellitus	39 (18%)	43 (25%)	0.14
	Family history of coronary artery disease	31 (15%)	32 (18%)	0.33
	Body mass index > 30 kg/m^2^	67 (32%)	40 (23%)	0.06
Previous history of coronary artery disease		2 (1%)	4 (2%)	0.29
Referral Source	Community	186 (88%)	112 (64%)	< 0.0001
	Hospital	10 (5%)	73 (42%)	< 0.0001

33% (70) of patients in cohort A and 45% (79) in cohort B underwent an echocardiogram. The most common indications for echocardiography were chest pain, dyspnoea and palpitations (Table 2).

**Table 2:** Indications for echocardiography

	Cohort A (n = 70)	Cohort B 2021 (n = 79)	Significance (p- value)
Chest pain	20 (29%)	54 (68%)	< 0.0001
Murmur/Valvular	8 (11%)	2 (3%)	0.03
Dyspnoea	17 (24%)	12 (15%)	0.16
Palpitations	10 (14%)	6 (8%)	0.19
Abnormal ECG/prior Echo	13 (19%)	2 (3%)	0.001
Other	2 (3%)	2 (3%)	0.90

**Fig. 1:**
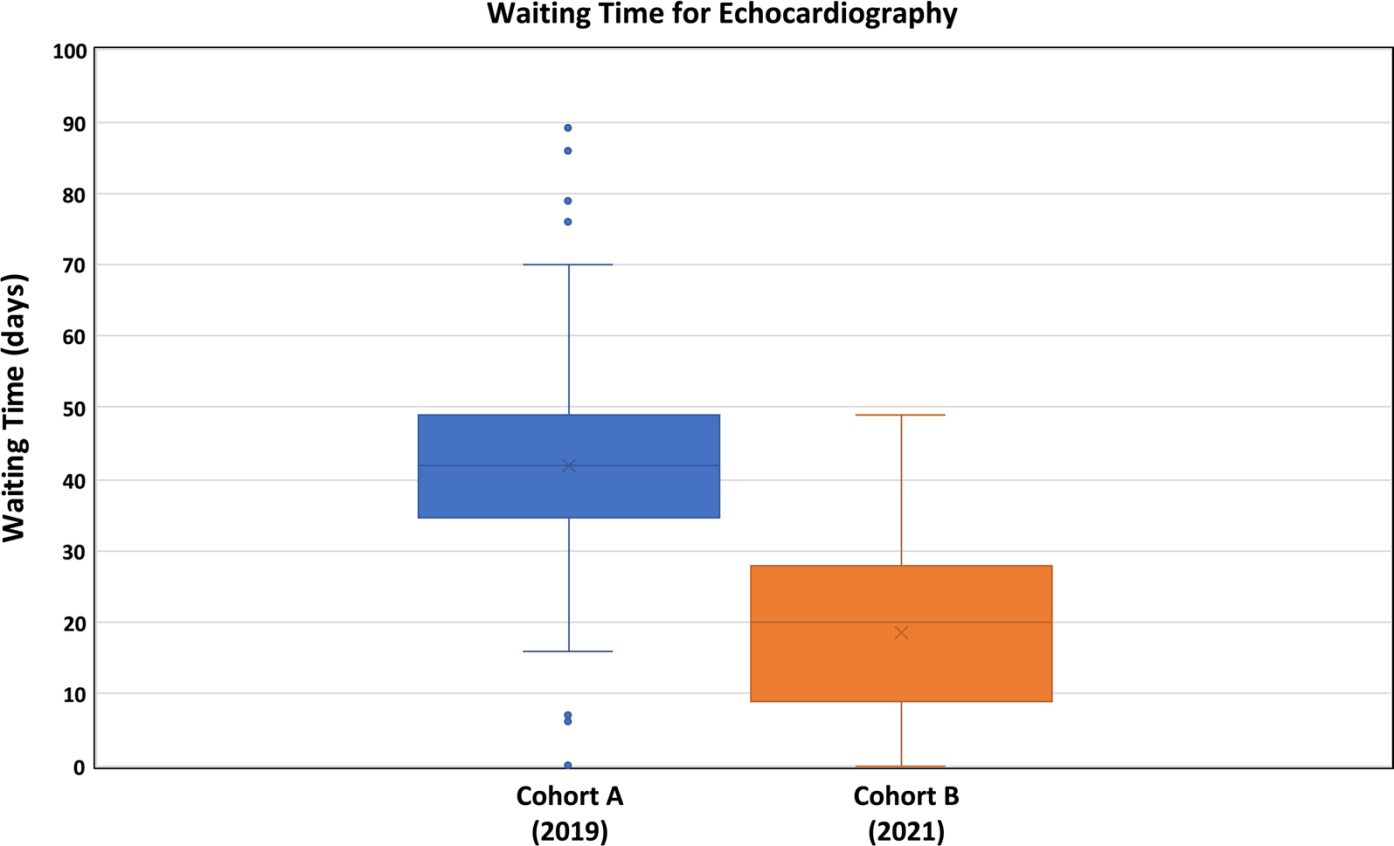
Median waiting time for echocardiogram (days) in cohort A (2019) and cohort B (2021)

The median waiting time for an echocardiogram following a RACP clinic appointment was significantly longer in cohort A as compared to cohort B [42 days (35–49 days) vs. 19 days (9–28 days); p < 0.00001] (Fig. 1).

**Fig. 2:**
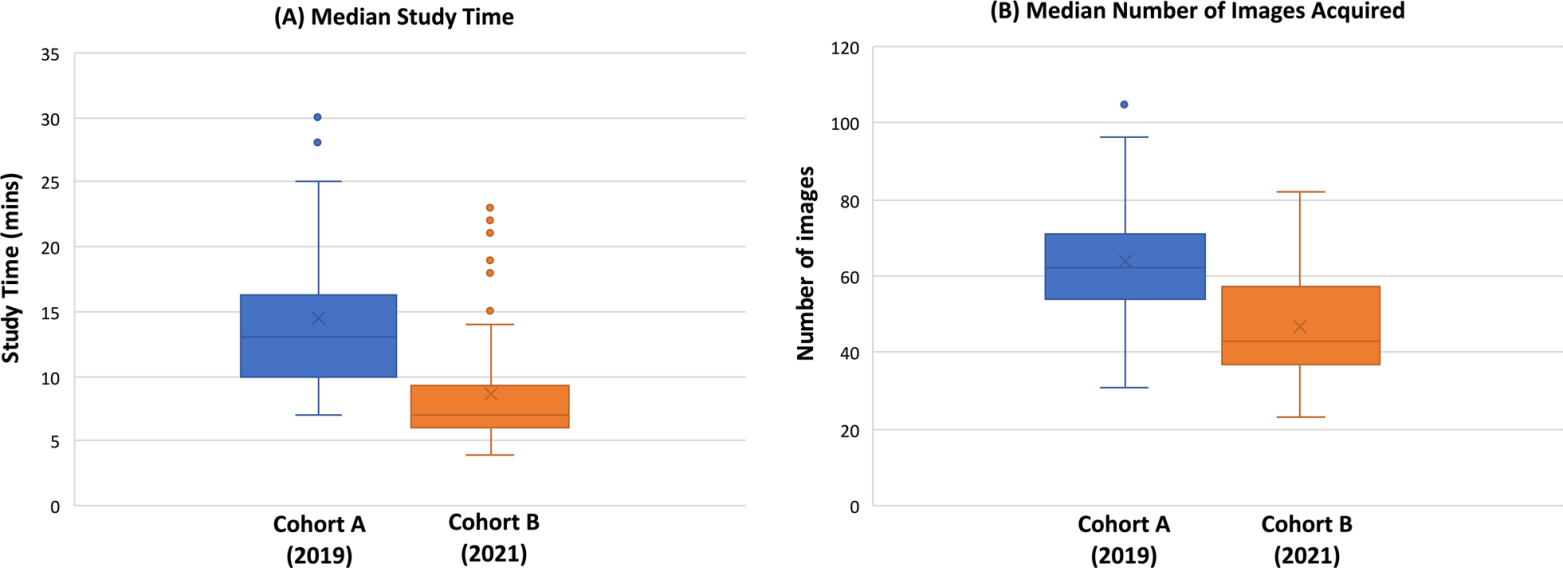
**A** Median echocardiogram acquisition time **B** Median number of images acquired per echocardiogram study

The median echocardiography study time was significantly longer in cohort A than cohort B [13 min (IQR: 10–16 min) vs. 7 min (IQR: 6–9 min), p < 0.00001], with a significantly higher number of images acquired in cohort A as compared to cohort B [median images: 62 (54–71) vs. 43 (37–57), p < 0.00001] (Fig. 2).

Abnormal echocardiograms were identified in 25 of the 70 patients (35.7%) in cohort A and 10 of the 79 (12.7%) in cohort B (p = 0.0009). These are summarised in Table 3. The most common echocardiographic abnormality in cohort A is diastolic dysfunction (11.4%). This was defined according to parameters outlined by the American Society of Echocardiography (ASE)/European Association of Cardiovascular Imaging (EACVI) guidelines, as adopted by the local echocardiography department. Excluding reported diastolic dysfunction, 17 (24.3%) patients were found to have abnormal echocardiograms in cohort A. 11 patients had valvular heart disease (15.7%; 4 mild, 6 moderate and 1 prosthetic valve).

**Table 3:** Echocardiography findings

	Cohort A (n = 70)	Cohort B (n = 79)	Significance (p-value)
Normal	45 (64.2%)	69 (87.3%)	0.0009
LV dysfunction	2 (2.9%)	3 (3.8%)	0.75
Regional wall motion abnormalities	3 (4.3%)	4 (5.1%)	0.82
Valvular heart disease (≥ mild)	11 (15.7%)	2 (2.5%)	0.004
Diastolic dysfunction	8 (11.4%)	0 (0%)	0.002
Pulmonary hypertension	3 (4.3%)	2 (2.5%)	0.55
Other	1 (1.4%)	2 (2.5%)	0.63

A significantly lower proportion of echocardiograms (12.7%) performed in cohort B showed abnormalities. The most common finding was regional wall motion abnormalities (5.1%), 2 of which were associated with normal overall LV systolic function.

There was concordance between the initial report and over-read on the grade of left ventricular function, presence and severity of any valvular disease and the presence or absence of pericardial effusion. There were discrepancies in three separate abbreviated echocardiography reports upon retrospective review. One was pertaining to the aetiology of mild valvular disease, another was regarding the degree of diastolic dysfunction and the final discrepancy was with regards to the presence of regional wall motion abnormalities in context of normal LV systolic function. No major pathology was missed in either cohort on re-read of all studies.

### Coronary assessment and outcome

Among patients referred for echocardiograms, 57% in cohort A and 78% in cohort B had concurrent coronary assessments. The majority of coronary assessments consisted of non-invasive imaging (computerised tomography coronary angiogram (CTCA): 34%; myocardial perfusion scan (MPS): 58%). 6% of the total study cohort undergoing echocardiograms were referred for a coronary angiogram. 2 patients in cohort A and 5 patients in cohort B declined coronary investigations.

Of those undergoing non-invasive coronary assessments, more than half in cohorts A and B had normal findings (cohort A: 53%, cohort B: 58%). Nearly a third (29%) of patients undergoing non-invasive ischaemia testing in cohort A had mild inducible ischaemia, as compared to 5% in cohort B. Of those undergoing CTCA, 14% of patients in cohort B were found to have mild coronary artery disease, as compared to 5% in cohort A.

Only 2 patients in cohort A (3%) underwent a coronary angiogram. Conversely, 7 patients in cohort B (9%) were referred for an invasive coronary investigation. 7 of 9 patients referred for a coronary angiogram underwent coronary revascularisation (6 percutaneous coronary interventions, 1 coronary artery bypass grafting). Of these, 4 had abnormal echocardiograms—3 with regional wall motion abnormalities, 2 of which also demonstrated left ventricular systolic dysfunction; 1 with mild aortic regurgitation. At the point of writing, there was one death among all patients in cohorts A and B. This was due to COVID-19 pneumonia.

## Discussion

This study demonstrates an abbreviated echocardiography protocol performed by level 2 physiologists is feasible in a low risk outpatient setting. Adoption of this protocol reduces acquisition time and increases available capacity. The protocol allows cardiovascular pathology to be quantified fully with good inter-observer agreement.

Cardiovascular ultrasound has evolved over the past decades. In an acute, inpatient setting, several focussed protocols have been adopted allowing basic assessment of a limited number of pathologies e.g. pericardial effusion or left ventricular dysfunction. These have shown high diagnostic accuracy with > 90% sensitivity [12, 13]. Whilst they have important clinical applications where rapid bedside assessment is necessary e.g. emergency medicine and critical care, these protocols have a limited scope of practice and a very focussed question. These studies can be performed on non-dedicated ultrasound machines without spectral Doppler by operators with limited echocardiography experience [14]. As a result, these protocols often lack a more detailed analysis of left ventricular wall motion abnormalities and quantification of valvular heart disease.

There are key differences between a focussed echocardiogram and a limited protocol. The American Society of Echocardiography (ASE) noted that the limited protocol has a wider scope of practice and is performed by level 2 or above physiologists or echocardiographers [8]. This means although the minimum number of images/views acquired is smaller than a full study, the more skilled physiologist is able to fully evaluate any pathology identified with additional views or measurements, when required. More recently, the ASE defined a set of limited echocardiography protocols relating to left ventricular function, pericardial effusion and pulmonary hypertension [10]. These protocols are often performed in patients with a prior full study and aim to provide adequate information focussed to a specific clinical question. Our clinical experience of low risk patients suggested a high proportion of normal echocardiograms. We therefore developed an abbreviated protocol for this patient group, with the potential advantage of tailoring echocardiography examinations to the need of the individual patients whilst improving access to echocardiography, through reduced overall scan time.

In this study, the majority of TTEs were performed in patients reporting chest pain, dyspnoea or palpitations. Interestingly, the RACP service in our centre has a relatively high echocardiography referral burden as compared to another UK centre (33–45% vs. 9.4%) [15–17]. This may suggest a co-morbid patient cohort with multiple cardiovascular risk factors, but also multifactorial, non-cardiac causes for breathlessness (e.g. obesity, 27.6% of study cohort) and chest pain. This is also consistent with international guidelines where these clinical symptoms would be considered appropriate indications for echocardiography according to both the ASE and the European Association of Cardiovascular Imaging (EACVI) [18, 19]. As such, this is an ideal cohort to introduce the abbreviated echocardiography workflow with the ability to fully evaluate any pathology.

Patients in cohort A had similar prevalence of major abnormalities on routine echocardiograms (35.7%) as compared to other observational studies performed in a selected cohort of patients with incidental heart murmurs. Conversely, cohort B demonstrated a significantly lower prevalence of major abnormalities on routine echocardiograms as compared these prior studies [16, 17]. Our protocol for abbreviated echocardiogram excluded patients with known valvular heart disease or structural heart disease. This may partly explain the discrepancy in abnormal findings between cohorts A and B, as well as between cohort B with other observational studies.

We also note a greater relative decrease in the median study time (46%) as compared to the number of echocardiography images acquired (31%). This is likely to reflect the lower level of complexity of studies performed under the abbreviated echocardiography protocol, possibly involving fewer quantitative assessments. In this study, we evaluated the clinical usefulness of each echocardiographic parameter to determine whether they should be included in the standard abbreviated protocol. We did not estimate left ventricular filling pressure as part of the standard protocol as the majority of pathologies associated with raised filling pressure e.g. hypertensive heart disease, aortic stenosis or left ventricular impairment would be identified and treated appropriately. However, physiologists were able to expand the dataset if abnormalities were encountered for example other signs of diastolic dysfunction including enlarged left atrium or pulmonary hypertension. In addition, abbreviated echocardiogram were not requested where the clinical likelihood of heart failure or valve disease was high.

Importantly, our study showed a significantly shorter average waiting time for echocardiograms of 14 days in 2021 vs. 42 days in 2019. In part, this is due to the improved workflow of being able to schedule 75% more abbreviated echocardiography slots in a session, facilitated by a 50% reduction in average scan acquisition time. Furthermore, previous studies have shown that the availability of limited or abbreviated echocardiography protocol does not increase the overall demand for TTEs over time [20]. This suggests there is a role of an abbreviated echocardiography protocol in improving the efficiency of echocardiography services, particularly in context of a low suspicion of cardiac abnormalities. The abbreviated echocardiography protocol may be especially helpful in context of an increasing number of virtual clinics and telephone consultations where physical examination of the patient is limited. This will be beneficial in reducing the waiting time for an already stretched echocardiography service and facilitate an efficient and cost-effective cardiology diagnostic pathway.

This study has several limitations. Firstly, the sample size is small and as such, this remains a pilot study. Further longitudinal studies detailing the long-term impact of abbreviated echocardiography protocols and correlation with patient outcomes are required. These findings are from a large single centre, tertiary hospital which may limit the applicability of the findings to other smaller centres. The population studied was a low-risk group with a high proportion of scans without significant pathology. Therefore, this data is unlikely to be applicable to patient cohorts with a high pre-test probability of abnormal echocardiographic findings, where the need to acquire more images would be required. In addition, the study has not accounted for other factors that may contribute to a reduced waiting time such as a lower echocardiography referral burden. However, the latter is less likely considering the ongoing service interruptions from the COVID-19 pandemic. The review or re-read of echocardiograms was performed retrospectively, and as such will not be able to identify pathology that was not captured when the scan was initially performed. Nevertheless, this is reflective of real-world echocardiography practice where scans performed by level 2 accredited physiologists or echocardiographers would not be routinely reviewed, unless specifically requested.

## Conclusion

In a low risk outpatient population, an abbreviated echocardiography protocol performed by level 2 or above physiologists significantly reduced waiting times through shortening the acquisition time, thereby increasing scheduling capacity and improving overall access to echocardiography services. This is achieved without compromising the diagnostic performance of echocardiograms performed. Further studies exploring the diagnostic performance of abbreviated echocardiography protocols and a cost–benefit analysis in different risk groups should be considered.

## Supplementary Information

**Additional file 1.** Recommended Minimum Dataset for Abbreviated Echocardiography.

## Data Availability

The datasets used and analysed during the current study are available from the corresponding author on reasonable request.
