# Supplementary material for: Feasibility, diagnostic performance and clinical value of an abbreviated echocardiography protocol in an out-patient cardiovascular setting: a pilot study
Source: Echo Res Pract. 2022 Sep 15;9:8. doi: 10.1186/s44156-022-00009-2 (PMC9473732; doi:10.1186/s44156-022-00009-2)
Supplement: Supplementary file 1 — Additional file 1. Recommended Minimum Dataset for Abbreviated Echocardiography. [file 44156_2022_9_MOESM1_ESM.docx]

**Recommended Minimum Dataset for Abbreviated Echocardiography**

| **Modality** | **Acquired images** | **Description** |
| --- | --- | --- |
| **Parasternal long axis** | | |
| 2D | Standard **PLAX** | For assessment of LV cavity size, wall thickness, radial systolic function, left atrium. |
| CFD | AV, MV | Assessment of AV regurgitation.  Assessment of MV regurgitation. |
| **Parasternal short axis** | | |
| 2D | Standard **PSAX** AV level | Assessment of valve morphology. |
| CFD | AV, TV | Assessment of AV, TV regurgitation. |
| ***CW* | *TV* | *If needed to assess pulmonary pressures.* |
| 2D  of basal LV | Standard PSAX MV level | Basal LV wall segment radial systolic function.  MV appearance. |
| 2D of mid LV | Standard PSAX Papillary muscle level | Mid LV wall segment radial systolic function and wall thickness.  Papillary muscle appearance. |
| 2D of apical LV | Standard PSAX Apex level | Apical LV wall segment radial systolic function and wall thickness. |
| CFD | MV | Assessment of MV regurgitation |
| **Apical 4-chamber** | | |
| 2D | Standard Apical 4 Chamber view | Assessment of LV systolic function and regional systolic function (Infero-septal and anterolateral walls).  Assessment of RV systolic function [mmode]  Assessment of MV and TV morphology.  Assessment of LA size. |
| CFD | MV, TV | Assessment of MV and TV regurgitation. |
| PW  CW | MV  TV | Assessment of MV inflow  TR jet |
| **Apical 5-chamber** | | |
| 2D | Standard Apical 5 Chamber view | Assessment of AV morphology. |
| CFD | AV | Assessment of AV regurgitation |
| ***CW* | *AV* | *If needed to assess AV stenosis* |
| **Apical 2-chamber** | | |
| *2D* | *Standard Apical 2 Chamber view* | *Assessment of LV systolic function and regional systolic function (Inferior and anterior walls).*  *Assessment of MV morphology.*  *Assessment of LA size* |
| *CFD* | *MV* | *Assessment of MV regurgitation.* |
| **Apical 3-chamber** | | |
| 2D | Standard Apical Long Axis view | Assessment of LV systolic function and regional systolic function (infero-lateral and antero-septal walls).  Assessment of MV and AV morphology. |
| CFD | AV, MV | Assessment of MV and/or AV regurgitation |
| **Subcostal** | | |
| 2D | Standard Subcostal 4 Chamber view  CFD | Assessment of pericardial effusion.  Assessment of interatrial septum integrity |
| 2D | IVC size | Assessment of IVC Size |

** Only if indicated

**Comparison between Minimum Dataset for Abbreviated Echocardiography Protocol vs. British Society of Echocardiography (BSE)-recommended Minimum Dataset**

|  | **Abbreviated** | **BSE-recommended** |
| --- | --- | --- |
| **PLAX (2D)**  **-LV cavity size and wall thickness**  **-LA (2D)**  **-CFD (AV and MV)**  **-Aorta (zoom)**  **-RVOT (incl. CW and PWD)**  **-RV inflow (incl. CWD)** | Y  Y   Y  Y  Only if indicated  Only if indicated  Only if indicated | Y  Y   Y  Y  Y  Y  Y |
| **PSAX (2D)**  **-Aortic valve level** *CFD*  ***Tricuspid Valve*** *CWD* ***RVOT*** *PWD*  **-Basal LV level** *Visual assessment of MV CFD*  *-***Mid ventricular** *Visual assessment* **-Apical** Visual assessment | Y Y  Only if indicated  Only if indicated  Y Y Y  Y   Y | Y Y  Y  Y  Y Y Y  Y  Y |
| **Apical 4-chamber**  Standard A4C view -*Assessment of LV and RV size and function (M-mode for TAPSE)*  -Assessment of LA size  MV and TV - *CFD*  MV Inflow - *PWD*  TV - *CWD*  -TDI (e’, E/e’, S’)  -PV identification incl. CFD and CWD)  -Modified A4C RV view | Y  Y  Y  Y  Y  Only if indicated  N  Only if indicated | Y  Y  Y  Y  Y  Y  Y  Y |
| **Apical 5-chamber**  Standard Apical 5-chamber  CFD  CWD  PWD | Y  Y  Only if indicated  Only if indicated | Y  Y  Y  Y |
| **Apical 2-chamber**  Standard Apical 2-chamber  CFD  Assessment of LA size | Y  Y  Y | Y  Y  Y |
| **Apical 3-chamber**  Standard Apical 3-chamber  CFD  CWD | Y  Y  Only if indicated | Y  Y  Y |
| **Subcostal**  Standard subcostal view  CFD  IVC assessment  Abdominal aorta assessment | Y  Y  Y  Only if indicated | Y  Y  Y  Y |
| **Suprasternal Notch**  Aortic arch assessment  CFD  CWD | Only if indicated  Only if indicated  Only if indicated | Y  Y  Y |

*(2D: 2-dimensional; CFD: Colour flow doppler; CWD: Continuous wave doppler; PWD: Pulse wave doppler; PLAX: Parasternal long-axis; PSAX: Parasternal short-axis; A4C: Apical 4-chamber; AV: aortic valve; MV: mitral valve; TV: tricuspid valve; PV: pulmonary vein; RVOT: right ventricular outflow tract; LV: left ventricle; RV: right ventricle; LA: left atrium; IVC: inferior vena cava; TAPSE: tricuspid annular plane systolic excursion; TDI: tissue doppler imaging )*
